# Coupling Coordination and Interactive Response Analysis of Ecological Environment and Urban Resilience in the Yangtze River Economic Belt

**DOI:** 10.3390/ijerph191911988

**Published:** 2022-09-22

**Authors:** Mei Yang, Mengyun Jiao, Jinyu Zhang

**Affiliations:** 1 School of Management, Chongqing University of Technology, Chongqing 400054, China; 2 Collaborative Research Center for Innovation-Driven Entrepreneurship, Chongqing University of Technology, Chongqing 400054, China

**Keywords:** ecological environment, urban resilience, coupling coordination, interactive response, the Yangtze River Economic Belt

## Abstract

There is a complex dynamic coupling interaction process between the ecological environment and urban resilience. It is important to clarify the coordination relationship and interactive response mechanism between them for sustainable development construction of the Yangtze River Economic Belt. The coupling coordination degree model and the panel vector autoregressive model (PVAR) were adopted to quantitatively examine the dynamic coordination and interactive response of the ecological environment and urban resilience in the Yangtze River Economic Belt from 2000 to 2019. Our study’s results are the following: (1) The ecological environment index and urban resilience index have a generally positive trend of fluctuation and increase during the study period but show significant regional differentiation. (2) The coupling coordination degree of ecological environment and urban resilience in the Yangtze River Economic Belt increased steadily, forming a spatial distribution pattern of “strong in the east and weak in the west”, with cities in the region mainly at the basic coordination level and generally lagging behind in development. (3) Both the ecological environment and urban resilience systems in the Yangtze River Economic Belt have significant self-reinforcing mechanisms, but the reinforcing effect is gradually decreasing, and the two positively promote each other, with urban resilience showing a more obvious promoting effect on the ecological environment.

## 1. Introduction

Cities exist in a symbiotic environment of nature and society. Cities integrate economy, social culture, ecological environment, and infrastructure, and their functions have attracted the attention of all countries in the world [1]. With the further advancement of global urbanization, the reduction in natural resources, the tense relationship between man and land, and the sharp loss of biodiversity have posed enormous challenges to human survival. Fortunately, more and more researchers are focusing on urban development and environmental protection in their investigations [2]. China is also facing the same problem in rapid urban development. China’s rapid economic development is also facing environmental pollution and ecological fragility [3]. These urban and environmental problems are not conducive to the implementation of China’s sustainable development strategy and may even be transformed into more severe black swan events. The Yangtze River originates in the Tibetan Plateau and flows through the densely populated eastern region of China [4]. The Yangtze River Basin spans 19 provinces (cities) in China from west to east, flows through Yunnan–Kweichow Plateau, the Sichuan Basin, the Middle and Lower Yangtze Valley Plain, and finally joins the East China Sea [5]. The Yangtze River Economic Belt is a major decision that China has made to promote national development. On the basis of promoting economic development and urban construction, the Yangtze River Economic Belt adheres to the concept of green development and pays attention to the protection of the ecological environment of the Yangtze River. How to balance high-quality urban development and high-level environmental protection is also a major problem facing the Yangtze River Economic Belt. Various natural disasters such as plagues, earthquakes and floods, and other crises, as well as social disasters such as terrorist attacks, extreme climates, and environmental pollution, are constantly testing the survival and development of cities [6], revealing numerous urban development challenges. Analysis of urban disaster preparedness methods from the perspective of vulnerability can reduce people’s exposure to hazards [7], while also addressing the lack of urban resilience. Urban resilience refers to the ability of cities to withstand risks, mitigate losses, and adapt to changes and recover, rebuild, and develop under foreseeable or unforeseeable risks, disaster shocks, and disturbances [8,9]. It helps cities reduce disturbances, prevent and resolve risks, and maintain or even surpass their previous level of development. Resilient cities are cities that have a high level of resilience and can resist, absorb, adapt, and respond effectively to hazards in a timely manner [10]. In 2013, the Rockefeller Foundation launched the “Global 100 Resilient Cities” project. Chinese cities such as Deyang in Sichuan and Yiwu in Zhejiang were selected in 2014 and 2016, respectively [11]. Beijing and Shanghai in China are the first to launch policies to build resilient cities. In the “Beijing Urban Master Plan (2016–2035)” and “Shanghai Urban Master Plan (2017–2035)”, they were officially released to enhance the city’s ability to cope with disasters. In 2018, the Disaster Prevention and Resilience for Urban and Rural Engineering, China Association for Disaster Prevention was established. In 2020, the Fifth Plenary Session of the 19th Central Committee of the Communist Party of China proposed for the first time to build a “resilient city”. Building a resilient city is officially written in China’s “14th Five-Year Plan” and the 2035 vision document. Cities are ever-changing systems that always rely on their natural ecological environment to maintain urban resilience and stability. The ecological environment not only includes the natural environment but also refers to the sum of the environments that are closely related to human beings and affect human life and production activities. The ecological environment is a complex ecological system related to human and urban social and economic development. The ecological environment can provide the material basis and various other necessary conditions for the survival and development of human beings [12]. Ecological environment is complex and fragile, and the development of cities will also significantly affect and change the natural ecological environment. In recent years, global climate change, frequent natural disasters, and “big city disease” have reduced the carrying capacity of urban ecosystems and caused self-adjusted imbalances, thus posing a serious challenge to the resilience and sustainable development of cities. The Ecological Civilization is one of multiple pathways to sustainable development, with Chinese characteristics to deal with China’s complex environmental problems in the process of development [2]. With the proposal of the Ecological Civilization, China’s cities and urban agglomerations have strengthened their investment in urban resilience. China not only coordinates the layout of production needs and living needs but also puts the ecological needs in a prominent position of sustainable development. It is necessary to solidify the ecological base of green development and focus on ecological environmental protection [13]. In the practice of building resilient cities in the Yangtze River Economic Belt, cities in the basin cannot implement large-scale development projects, which is an important opportunity to find new paths for environmental protection and urban development. Therefore, what is the coordinated relationship between the ecological environment and urban resilience? How do they interact and to what extent do they contribute to each other? These issues require quantitative analysis and empirical research. Our study provides data support and case samples for theoretical studies and governance decisions, which can guide the coordination and interaction between the ecosystem and urban resilience. At present, the academic community has initially formed an urban resilience assessment system including ecology, engineering, society, and economy through research on countries, urban agglomerations, and regions. They pay attention to the interrelationship between urban resilience and systems such as economic development level [14], urbanization level [15], land development intensity [16], and science and technology innovation [17]. In the interactive relationship between ecological environment and cities, the relationship between the ecological environment and economic growth [18], tourism economy [19], urbanization [20], and economic society [21] is the research hotspot. However, the coupling coordination analysis between ecological environment and urban resilience is relatively lacking.

The Yangtze River Economic Belt covers 11 provinces and cities, including Shanghai, Jiangsu, Zhejiang, Anhui, Jiangxi, Hubei, Hunan, Chongqing, Sichuan, Yunnan, and Guizhou. It is an inland economic belt with global influence and a pioneer demonstration belt for ecological civilization construction [22]. Adhering to the ecological priority and green development of the Yangtze River Basin is an important foundation and prerequisite for building the Yangtze River Economic Belt. The Yangtze River Economic Belt not only pays attention to environmental protection but also is a pioneer area for China’s resilience building. Therefore, taking the Yangtze River Economic Belt as an example, we scientifically measure the interactive response relationship and deeply analyze their mechanism between the ecological environment and urban resilience. This can help clarify the current situation of coordination between urban resilience and environmental protection in China and solve the development problems faced by countries around the world. Our research can alleviate the relationship between man and land to a certain extent and provide a new perspective for the balance between the city and the environment, as well as provide inspiration and reference for other countries’ resilient city construction and ecological environmental protection through China’s experience. This study takes the Yangtze River Economic Belt as the research object, constructing a comprehensive evaluation index system of ecological environment and urban resilience based on the panel data from 2006 to 2020. Firstly, the entropy method was used to calculate the ecological environment index and urban resilience index. Secondly, the coupling coordination degree model was employed to reveal the coupling coordination mechanism of the two. Finally, the panel vector autoregressive estimation model was conducted to investigate the interactive response relationship between the ecological environment and urban resilience.

## 2. Theoretical Framework and Coordination Mechanism

### 2.1. Theoretical Basis: Sustainable Development Theory

After World War II, the global socio-economic development concept underwent a major transformation process from “growth theory” to “development theory” and then to “sustainable development theory” [23]. In 1987, the concept of “sustainable development” was clearly put forward after the publication of “Our Common Future” by the World Commission on Environment and Development. After the “Agenda 21” was signed in 1992, the sustainable development strategy was implemented into global action and practice for the first time [24]. In 2000, 189 countries adopted the United Nations Millennium Declaration, committing to creating an environment conducive to development and poverty eradication for the world [25]. In 2015, at the United Nations Sustainable Development Summit, the “Transforming our World—the 2030 Agenda for Sustainable Development” was adopted and the Sustainable Development Goals (SDGs) were listed [26]. From 1992 to 2015, the connotation of sustainable development was continuously enriched. However, the core of sustainable development has always emphasized the three bottom lines of economy, social culture, and environment [27]. The global socio-economic development concept no longer focuses only on economic growth but also on the process of economic, environmental, and social development [28]. At different stages of global development, these three aspects are constantly adjusted, shifting from economic development to social-development-led ones. The social-development-led concept of sustainable development emphasizes the view of development from the perspective of social well-being [29]. Based on the experience of the global sustainable development goals, we conduct research on localization in China. At the United Nations Sustainable Development Summit in 2002, the concept of resilience was first introduced into the field of urban planning and disaster prevention, setting off a research upsurge of “urban resilience” in China. The core of sustainable development at this stage emphasizes the three bottom lines of economic, socio-cultural, and environmental development [27], so it is necessary to take into account economic responsibility, social responsibility, and environmental responsibility at the same time. Current urban development should not only strengthen resilience thinking and resilience concepts but also emphasize the central role of the environment and bottom-line thinking [30]. The essence of sustainable urban development has three obvious characteristics of development, coordination, and sustainability [31]. It provides important theoretical support for the coordination mechanism and response mechanism of the ecological environment and urban resilience in this study. Firstly, we calculate the level of the ecological environment index and the urban resilience index. We emphasize that the ecosystem and the urban system are gradually healthy and constantly reach high-level and high-quality development through the “development degree”. Secondly, we take advantage of the coupling and coordination effect of the two. We emphasize the interdependence, synergy, and harmonious development between systems through the “coordination degree”. Finally, the study of the interactive response relationship between ecological environment and urban resilience is the embodiment of the “sustainability degree”. It is based on the long-term regulation mechanism of ecological environment and urban resilience, focusing on the “time or process” of the two to grasp the “development degree” and the “coordination degree” [32].

**Figure 1:**
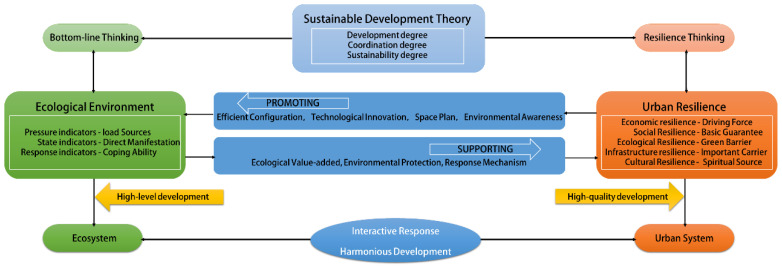
A framework for the coordination mechanism of ecological environment and urban resilience.

In conclusion, the coordination mechanism between ecological environment and urban resilience needs to explore the interdependence in terms of development level (development degree), coordination relationship (coordination degree), and future contribution (sustainability degree). These three aspects jointly contribute to the sustainable development capacity and construction of the Yangtze River Economic Belt. Therefore, this study takes the interrelationship between ecological environment and urban resilience as the main body and constructs the analytical framework of their interaction mechanism (Figure 1).

### 2.2. The Mechanism of Ecological Environment on Urban Resilience

A pleasant ecological environment can promote the stable operation of cities and the orderly advancement of urbanization. It can also deal with the premise and guarantee of capacity building for various natural and social disasters, that is, the ecological environment has an important supporting role for urban resilience. The specific action path is manifested in three aspects: (1) Give full play to the ecological value-added function of the ecological environment and bring material bases and value elements to urban resilience. The statement “Clear waters and green mountains are as good as mountains of gold and silver” provides new ideas for the transformation of ecological product value [32]. Clear waters and green mountains are not only natural wealth and ecological wealth but also social wealth and economic wealth [33]. A pleasant ecology itself contains infinite economic value and can create comprehensive benefits for urban development. By taking advantage of natural advantages to develop characteristic industries, a market-oriented and diversified ecological compensation mechanism can be established to promote a more resilient and sustainable urban system. (2) Environmental conditions provide a guarantee for urban resilience. A pleasant ecological environment can provide a variety of ecosystem services, alleviate the conflict between man and land, and provide protection for cities against various shocks such as climate change, energy shortage, and financial turmoil. Protecting and restoring the ecological environment is the main line of work in the Yangtze River Basin. By breaking the barriers between urban resources, information can be fully circulated between various departments and cities, thereby shaping the overall improvement pattern and comprehensive resilience governance mechanism. (3) Actively build an ecological response mechanism to improve urban resilience disaster response. The construction of ecological response mechanism includes disaster early warning information platforms, risk prevention and control emergency management, etc. It is not limited to the administrative or geographical boundaries of individual city systems but integrates urban ecological security and human settlement environment health at the scale of the river basin to achieve new breakthroughs in decision making and early warnings of urban resilience.

### 2.3. The Mechanism of Urban Resilience on the Ecological Environment

The high-quality development of urban resilience can accelerate the restoration of the ecological environment, enhance the stability of ecosystem, and improve the self-mediation ability of the ecological environment to cope with disasters and shocks, that is, urban resilience construction is an important driving force for ecological environmental protection and ecological restoration. The specific action path is manifested in four aspects: (1) Realizing efficient resource allocation and relieving the burden of environmental carrying capacity. Urban resilience coordinates multi-sectoral and multi-factor relationships and improves the efficiency of factor allocation. By optimizing the industrial structure, it promotes the orderly development of economy, society, ecology, culture, and infrastructure. Therefore, the environmental pressure is effectively relieved. (2) Urban resilience construction guides cities to carry out technological innovation and key technology research. The Yangtze River Basin attaches great importance to basic research on the causes and processes of environmental pollution, health impact mechanisms, and risk assessment of environmental pollutants [34]. Achieve the maximum effect of governance with the least economic cost, promote scientific and technological innovation as a new driving force for green development, and realize scientific and technological innovation to empower the ecological environment. (3) Rational development and planning. Resilient cities focus on ecological protection, water system layout, functional zoning, municipal and transportation infrastructure, and other construction. Meanwhile, adhere to the new development philosophy of innovation, harmonization, green, openness, and sharing, integrated in the prediction, protection, and management of disaster risks, and optimize the layout of urban and ecological space. (4) Resilient cities are constantly being constructed and updated, and the focus and strength of their humanistic systems and ecological environment systems are also continually changing. With the continuous strengthening of cultural and social resilience, urban residents’ awareness of environmental protection has gradually increased. The participation in environmental protection has been enhanced, which helps promote the synergistic governance and development of ecological and urban systems.

## 3. Materials and Methods

### 3.1. Ecological Environment Index (EE) and Urban Resilience Index (UR)

#### 3.1.1. Construction of Evaluation Index System

Based on the analysis of the coordination mechanism of ecological environment and urban resilience, their evaluation index system is constructed. (Table 1).

Regarding the EE evaluation model, the Pressure–State–Response (PSR) framework model is selected to reflect the interaction relationship between urban systems and ecosystems [35]. This study constructs the index system from three aspects: pressure indicators, state indicators, and response indicators, and it contains 12 indicators in total. (1) pressure indicators are the load sources of EE, and four key indicators are selected, such as natural disasters, industrial wastewater, industrial waste gas, and agricultural pollution; (2) state indicators are the direct manifestation of the background state of EE, and four key indicators are selected, such as air, water resources, forests, and green areas; (3) response indicators are the coping ability of EE, and four key indicators are selected, such as soil erosion control, environmental protection expenditure, garbage removal and transportation, and sewage treatment.

Regarding the evaluation model of the UR, the existing literature mainly constructs the evaluation framework from the perspective of economy, society, ecology, and infrastructure [36,37]. These four aspects are the UR model and assessment basis recognized by many scholars. This shows that urban resilience is a process of comprehensive development of multiple factors. Based on the four dimensions of ecological, economic, social, and infrastructure resilience, we added the dimension of cultural resilience. The cultures of the Yangtze River Basin, such as the Jianghuai culture, the Bashu culture, and the Hakka culture, have both sameness and diversity and play an important role in the history of the development of Chinese civilization. In the process of sustainable development, many well-intentioned projects are hindered by a lack of identification with local culture and values [38]. Culture offers new ideas for sustainable development and guides our attention to regional value systems, traditions, and beliefs [39]. At the same time, culture is the soft power of urban development and an important source of the UR [40]. Therefore, we constructed an indicator system from five aspects: economic, social, ecological, infrastructure, and cultural resilience, including a total of 30 indicators. (1) Economic resilience is the ability of urban economies to maintain a stable economic environment and economic development under the impact and pressure of internal and external uncertainties and is the driving force of the UR [41]. Six key factors, such as GDP per capita and local public revenue, are selected as indicators of economic resilience. (2) Social resilience is the ability of cities to resist risks, operate in an orderly manner, and improve quality and efficiency and is the basic guarantee of the UR. Six key factors such as population density and regional urbanization rate are selected as indicators of social resilience. (3) Ecological resilience is the ability of the urban ecological environment to resolve the impact of natural disasters and social pollution to the greatest extent [42], and it is the green barrier for the UR. Six key factors such as forest coverage rate and harmless treatment rate of domestic waste are selected as the indicators of ecological resilience. (4) Infrastructure resilience is the ability of urban infrastructure to resist, absorb, and recover to normal state under internal and external shocks [43], which is an important carrier of the UR. Six key factors such as per capita electricity consumption and per capita urban road area are selected as the characterization indicators of infrastructure resilience. (5) Cultural resilience is the ability of a city to demonstrate cultural identity and cultural cohesion even after being impacted, and it is the spiritual source of the UR. Six key factors, such as the proportion of public cultural services and the number of employees in cultural, sports, and entertainment industries per 10,000 people, are selected as indicators of cultural resilience.

#### 3.1.2. Comprehensive Index Evaluation Method

Regarding the calculation of the comprehensive index of the EE and UR, in order to avoid the influence of bias caused by subjective factors, the entropy method is applied to determine the weights of the two systems [44]. The entropy method is a method to determine the weights based on the information entropy of the index data, where the greater the information entropy, the greater the amount of information provided, and the greater the weight of the index [45]. According to the determined weights, this study uses the linear combination method to evaluate the comprehensive index of the EE and the comprehensive index of the UR. The equation of the entropy weight method is as follows:

Data standardization:

(1) $$\{\begin{array}{c} x_{ij}^{*}=\frac{x_{ij}-\min\left(x_{ij}\right)}{\max\left(x_{ij}\right)-\min\left(x_{ij}\right)}+0.0001\;Positive\;indicator\\ x_{ij}^{*}=\frac{\max\left(x_{ij}\right)-x_{ij}}{\max\left(x_{ij}\right)-\min\left(x_{ij}\right)}+0.0001\;negative\;indicator \end{array}$$

where $x_{ij}^{*}$ represents the standardized index value. $x_{ij}$ represents the initial value of the data. $\mathrm{Max}\left(x_{ij}\right)$ represents the maximum value of the initial value of the data. $\min\left(x_{ij}\right)$ represents the minimum value of the initial value of the data. i represents the ith evaluation object, and j represents the *j*th index in the evaluation system.

Determine the weights:

(2) $$w_{j}=\frac{1-[-k\sum_{i=1}^{n}\frac{x_{ij}^{*}}{\sum_{i=1}^{n}x_{ij}^{*}}\ln\left(\frac{x_{ij}^{*}}{\sum_{i=1}^{n}x_{ij}^{*}}\right)]}{\sum_{j=1}^{m}\left\{1-[-k\sum_{i=1}^{n}\frac{x_{ij}^{*}}{\sum_{i=1}^{n}x_{ij}^{*}}\ln\left(\frac{x_{ij}^{*}}{\sum_{i=1}^{n}x_{ij}^{*}}\right)]\right\}},\;k=\ln\frac{1}{n}$$

(3) $$w_{j}^{*}=\frac{\sum_{t=1}^{z}w_{j}}{z}$$

where $-k\overset{n}{\underset{i=1}{\sum }}\frac{x_{ij}^{*}}{\sum_{i=1}^{n}x_{ij}^{*}}\ln\left(\frac{x_{ij}^{*}}{\sum_{i=1}^{n}x_{ij}^{*}}\right)$ represents the entropy of the *j*th indicator. $1-[-k\overset{n}{\underset{i=1}{\sum }}\frac{x_{ij}^{*}}{\sum_{i=1}^{n}x_{ij}^{*}}\ln\left(\frac{x_{ij}^{*}}{\sum_{i=1}^{n}x_{ij}^{*}}\right)]$ represents the difference coefficient of the *j*th index. n represents the number of evaluation objects. m represents the number of indicators in the evaluation system. z represents the length of the research period.

Evaluation:

(4) $$EE=w_{j}^{*}\times \overset{n}{\underset{i=1}{\sum }}x_{ij}^{*}$$

(5) $$UR=w_{j}^{*}\times \overset{n}{\underset{i=1}{\sum }}x_{ij}^{*}$$

where EE represents the ecological environment index. UR represents the urban resilience index. $w_{j}^{*}$ represents the weight corresponding to the *j*th indicator. $x_{ij}^{*}$ represents the standardized indicator value.

### 3.2. Coupling Coordination Degree Model

Coupling refers to the phenomenon that two or more systems affect each other through interaction, and thus produce dynamic association [46]. Since the coupling degree model has difficulty fully reflecting the degree of coordinated development between systems [47], this study introduces the coupling coordination degree model to reflect the coupling coordination relationship of the EE and UR. By drawing on the treatment of the coupling coordination degree model [48], the coupling coordination degree equation in this study is as follows:

(6) $$C={\left[\frac{EE\times UR}{{\left(\frac{EE+UR}{2}\right)}^{2}}\right]}^{\frac{1}{2}}$$

(7) T=αEE+βUR

(8) $$D=\sqrt{C\times T}$$

where *EE* and *UR*, respectively, represent the ecological environment index and urban resilience index; α  and β are undetermined coefficients. Considering that ecological environment and urban resilience are equally important in the overall system, the values α = 0.5 and β = 0.5 are assigned; C represents coupling degree; *T* represents comprehensive coordination index; and D  represents coupling coordination degree. The larger the value of D, the more coordinated the coupling relationship between ecological environment and urban resilience. Meanwhile, the coupling coordination degree was divided into 4 types and 12 characteristics by considering the coordination level classification of scholars such as Wang [49] and Gan [50] (Table 2).

### 3.3. The Panel Vector Autoregressive Model (PVAR)

Considering the complex mechanism of action between the EE and UR systems, the use of ordinary regression models will face problems such as endogeneity and heteroskedasticity [32]. The panel vector autoregressive models (PVAR) combine the advantages of the autoregressive models (VAR) and panel data, treating all variables as endogenous variables. By introducing different individual effect and time effect variables at the same time, the impulse response analysis is applied to analyze the impact of one endogenous variable on other endogenous variables, which can reflect the problem of individual heterogeneity and the common shocks received by different sections [51]. The panel vector autoregressive models can effectively consider the dynamic relationship between variables. Therefore, this study uses the PVAR to explore the interactive response relationship between ecological environment and urban resilience in Yangtze River Economic Belt. The PVAR model is set as follows.

(9) $$y_{it}=\alpha_{i}+\beta_{0}+\sum_{k=1}^{l}\beta_{k}y_{i,t-k}+\lambda_{it}+\varepsilon_{it}$$

where *yit* represents the two-dimensional column vector of the logarithm value of the ecological environment index (lnEE) and the logarithm value of the urban resilience index (lnUR) in the i region in year *t*. $\alpha_{i}$ represents the individual fixed effect vector.$\;\lambda_{it}$ represents the time-fixed effect vector. $\beta_{0}$ represents the intercept term vector. $\beta_{k}$ represents the parameter matrix of the lagged variables. $\varepsilon_{it}$ represents the random disturbance term. i represents the region.  t represents the year. k represents the lag order.

### 3.4. Data Sources

The Yangtze River originates from the Qinghai–Tibet Plateau, with a total length of 6300 km, spanning the three major economic regions of eastern, central, and western China. The total area of the Yangtze River Basin is 1.8 million square kilometers, accounting for 18.8% of China’s land area. The basin is rich in natural resources. The Yangtze River Economic Belt includes 11 provinces (cities) in China. Among them, the upper reaches of the Yangtze River include four provinces and cities: Chongqing, Sichuan, Yunnan, and Guizhou. The middle reaches of the Yangtze River include three provinces and cities: Jiangxi, Hubei, and Hunan. The lower reaches of the Yangtze River include Shanghai, Jiangsu, Zhejiang, and Anhui provinces and cities. This paper takes the 2006–2020 period as the research period and selects 11 provinces and cities in the Yangtze River Economic Belt for empirical analysis. The required data came mainly from the “China City Statistical Yearbook (2007–2021)”, “China Statistical Yearbook (2007–2021)”, and the statistical documents published by the statistical bureaus of the regions in the Yangtze River Basin. For individual missing data, the mean substitution method was used to supplement.

## 4. Results

### 4.1. Analysis of Coupling Coordination Degree between the EE and UR

Firstly, Equations (1)–(5) were used to evaluate the EE and UR of 11 provinces and cities in the Yangtze River Economic Belt from 2006 to 2020. Then, Equations (6)–(8) were used to calculate the coupling coordination degree of the EE and UR. Considering that the EE and UR face various pressures and shocks, as well as the replacement of the evaluation standard of excellent air quality rate, it is easy to cause data fluctuations and cause large errors. Therefore, this study selected the three-year average method. We divided the 2006–2020 period into five research periods, namely Period one (2006–2008), Period two (2009–2011), Period three (2012–2014), Period four (2015–2017), and Period five (2018–2020). This section may be divided by subheadings. It should provide a concise and precise description of the experimental results, their interpretation, as well as the experimental conclusions that can be drawn.

#### 4.1.1. Ecological Environment Index (EE) and Urban Resilience Index (UR)

**Figure 2:**
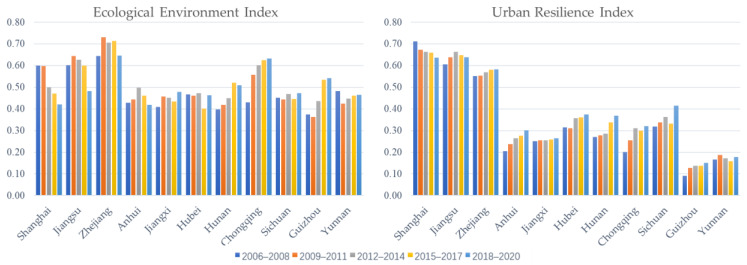
Time characteristics of the ecological environment index and urban resilience index.

(1) Time characteristics. Figure 2 shows the EE and UR of most provinces and cities in the Yangtze River Economic Belt fluctuated and increased during the 2006–2020 period, indicating a good overall development trend of the ecological environment and urban resilience level of the Yangtze River Economic Belt. Fifteen years of long-term structural adjustment, green adjustment of production links, continuous strengthening of urban hardware systems, and empowerment of scientific and technological innovation has brought high-quality performance of stable growth to the overall level of the EE and UR.

Firstly, we analyze the EE in detail. The overall performance of Shanghai is declining, and this trend may be related to Shanghai’s “big city disease” as a mega city. Although there are contradictions in Shanghai’s urban spatial pattern, public service supply, and transportation corridor construction due to the continuous promotion of the high-level protection of the ecological environment, the EE has been maintained at a high level. Jiangsu, Zhejiang, and Anhui show a change in the form of first rising and then falling, among which Zhejiang has the highest EE, always above 0.64. Since 2006, Zhejiang has practiced “Clear waters and green mountains are as good as mountains of gold and silver”, always demonstrating the determination of green development. Jiangxi, Hunan, Chongqing, and Guizhou have seen a steady increase in the EE, with Chongqing and Guizhou showing the most prominent increase, reflecting that the provinces and cities in the middle and upper reaches of the Yangtze River put the restoration of the ecological environment of the Yangtze River in an overwhelming position.

Secondly, the overall change in the UR is small and relatively more stable, but the UR is generally low. Shanghai’s UR decreased over 15 years, but the overall average value is 0.67, which is still much higher than other regions, indicating that Shanghai’s resilient city construction has achieved remarkable results and has a higher ability to survive, recover, and adapt in terms of urban risk prevention. The URs of Jiangsu, Sichuan, and Yunnan show a rise first and then a decline, with Sichuan and Yunnan experiencing a rapid decline in the urban resilience index of Period 4 compared with the previous period. The URs of Jiangsu, Sichuan, and Yunnan showed an increase first and then a decrease. Among them, the resilience indices of the four cities in Sichuan and Yunnan declined rapidly compared with the previous period. The reason may be that during the “12th Five-Year Plan” period in Jiangsu, natural disasters were frequent. Sichuan and Yunnan suffered from 3.25 wind and hail disasters, floods, continuous debris flows, and increased earthquake risks during the 2015–2017 period. The URs of Jiangsu, Sichuan, and Yunnan have been significantly reduced due to disasters in successive years. Other provinces and cities, such as Zhejiang, Anhui, Jiangxi, Hubei, and Chongqing, show an upward trend in the urban resilience index, but the magnitude of the increase has some differences.

**Figure 3:**
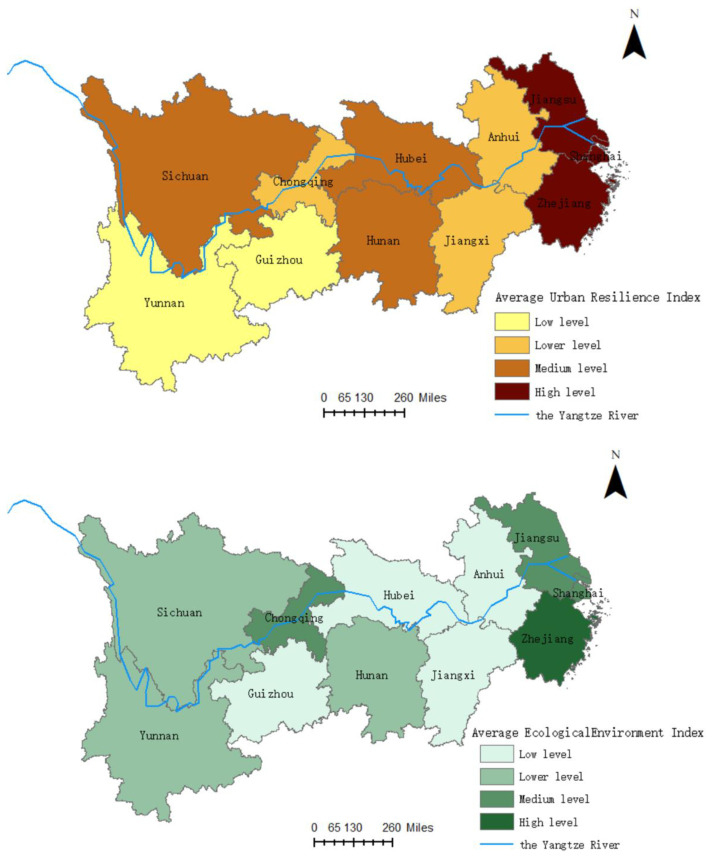
Spatial distribution of the average value of the ecological environment index and mean value of urban resilience.

(2) Spatial characteristics. We calculated the average values of the EE and UR from 2006 to 2020 and divided them into four levels using the natural breakpoint classification method of ArcGIS 10.7 software (Environmental Systems Research Institute, Redlands, CA, USA) (Figure 3). Both the EE and UR of the Yangtze River Economic Belt show significant regional differences, with greater regional differences in urban resilience.

The average value of the ecological environment index of the Yangtze River Economic Belt generally presents a spatial distribution pattern of “lower reaches of the Yangtze River > upper reaches of the Yangtze River > middle reaches of the Yangtze River”. The lower reaches of the Yangtze River have strong comprehensive strength. With the implementation of environmental pollution control and siltation transportation and other measures, the ecological environment has been significantly improved, with the average values of the EEs of Zhejiang, Jiangsu, and Shanghai reaching 0.69, 0.59, and 0.52, respectively, which are at a high and medium level. The highest mean value of EE in the upper reaches of the Yangtze River is Chongqing, with a value of 0.57, which is at a medium level of ecological environment. Sichuan and Yunnan have a value of 0.46, which is at a lower level of ecological environment, and Guizhou has the lowest ecological environment index, which is at a low level. This is mainly due to the high ecological vulnerability and frequent natural disasters in the upper reaches of the Yangtze River, hence the poor performance of the index level. The provinces in the middle reaches of the Yangtze River are all at the low and lower levels of the EE, and it is necessary to focus on improving the quality and efficiency of ecological environmental protection.

The regional differences in the mean value of the UR are more significant, with a spatial distribution pattern of “lower Yangtze River > middle Yangtze River > upper Yangtze River” in general. Except for Anhui, all cities in the lower reaches of the Yangtze River are at a high level of urban resilience, and the spatial distribution is more concentrated, which is conducive to the extensive coverage of resilience among regions and the formation of regional linkage and synergy. Hunan and Hubei, in the middle reaches of the Yangtze River, are at 0.30 and 0.34, respectively, both in the middle level of urban resilience, while Jiangxi’s resilience level is lower at 0.27, which is at a lower level of urban resilience. In the upper reaches of the Yangtze River, Sichuan has the highest average UR at 0.35, which is at a medium level of urban resilience, followed by Chongqing, which is at a lower level of urban resilience, while Yunnan and Guizhou have the lowest urban resilience indexes at 0.17 and 0.13, respectively. It shows that the construction of resilient cities in the upper reaches of Yangtze River is weak, the resilience capacity is insufficient, and there is a large gap with the middle reaches of the Yangtze River and lower reaches of the Yangtze River. From the perspective of individual cities, the current resilience level of the Yangtze River Economic Belt is still low, except for Shanghai, Zhejiang, and Jiangsu. Therefore, the construction of urban resilience is an important direction for the future planning of the Yangtze River Economic Belt. It is necessary to effectively strengthen regional synergistic development, shrink the differences between regions and enhance the overall effect, strengthen resilience management, and realize the overall improvement of resilience level in the basin.

#### 4.1.2. Coupling Coordination Degree between the EE and UR

**Figure 4:**
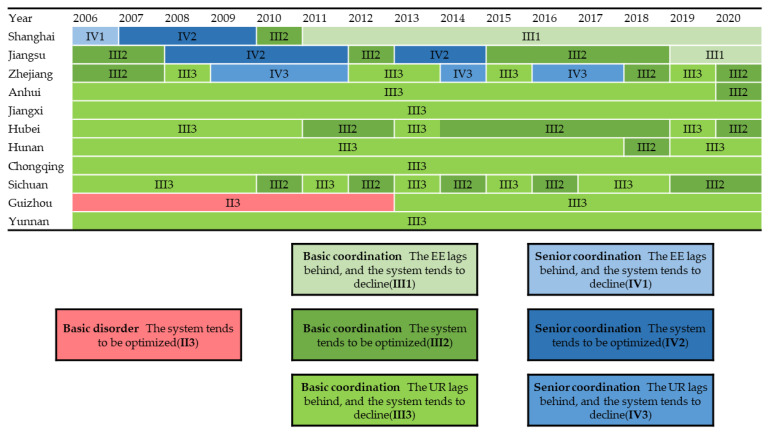
Characteristics of Coupling Coordination between the EE and UR.

Based on the evaluation of the EE and UR, the coupling coordination degree of the EE and UR of each province and city in the Yangtze River Economic Belt from 2006 to 2020 was calculated by using the coupling coordination degree model (Table 3). Additionally, according to Table 2, we divided the coordination level, and the results are shown in Figure 4. Overall, the coordination level of the EE and UR in the Yangtze River Economic Belt has steadily increased over the past 15 years, forming a spatial distribution pattern of “strong in the east—weak in the west”. In terms of coordination level classification, the coupling coordination degree is mainly at the basic coordination level and above, and the coordination is in good condition. Meanwhile, the lower reaches of the Yangtze River generally show the lagging characteristics of the EE, and the middle and upper reaches of the Yangtze River show the lagging characteristics of the UR.

As the leading city in the development of Yangtze River Economic Belt, Shanghai has an average of 0.77 for the coupling coordination degree. During the study period, it achieved a negative leap from senior coordination to basic coordination and is mainly characterized by ecological environment lag. This indicates that the main factor inhibiting the improvement of coordination in Shanghai is the lack of eco-environmental dynamics. The mega city Shanghai shoulders the heavy responsibility of economic development, emphasizing the quality and efficiency of economic development, while focusing on the agglomeration of social, financial, trade, and innovation linkages and the relative lack of ecological environmental development power. The coupling coordination degree average of Zhejiang and Jiangsu are 0.79 and 0.78, respectively, which are in the basic coordination type. Additionally, the coordination characteristics of Zhejiang mainly show the urban resilience lagging type, while Jiangsu’s EE and UR promoted each other during the 2006–2018 period, and the system tends to be optimized, and it is stable and maintained in the advanced coordination stage for four consecutive years from 2008–2011, while the 2019–2020 period shows an ecological environment lagging type, indicating that the main reason limiting the further improvement of coupled coordination in Zhejiang and Jiangsu is also the relatively weak development of ecological environment, and therefore remains in the transition stage of basic coordination–advanced coordination for a long time. The mean values of the coupling coordination degree in Anhui, Jiangxi, Hubei, Hunan, Chongqing, Sichuan, and Yunnan regions are 0.58, 0.58, 0.63, 0.61, 0.63, 0.63, and 0.53, respectively, which are always in the basic coordination stage during the study period and show the urban resilience lag type. This demonstrates that urban resilience lags behind ecological environment development, and the two are poorly coordinated, and the system tends to decline. Among them, Hubei and Sichuan have relatively good system characteristics. For a period of time, urban resilience is synchronized with the development of the ecological environment. Therefore, the level of coupling coordination is higher than other regions. Chongqing always manifests basic coordination and urban resilience lag type during the study period, but the level of the coupling coordination is generally higher than that of other regions of the same type. This indicates that the Chongqing region, relying on the driving effect of the ecological environment, actively guides the stable growth of urban resilience level, compensates for the lack of urban resilience dynamics more quickly, gradually improves urban warning, recovery, and adaptive capacity, and promotes the coordinated and orderly development of both. The coupling coordination degree in Guizhou during the 2006–2012 period is less than 0.5 and is at the basic disorder stage, while the coupling coordination degree during the 2013–2020 period is between 0.5 and 0.53, steadily upward to the basic coordination stage. This may be due to the urban resilience of Guizhou always lagging behind ecological environment development and being constrained by the urban resilience level. In short, the lower reaches of the Yangtze River are generally characterized by the ecological environment lag type, while the middle and upper reaches of the Yangtze River are characterized by urban resilience lag type. Both rely on a single system to maintain the coordinated development of the two, and the overall system tends to decline, which is not conducive to the sustainable development of the Yangtze River Economic Belt.

### 4.2. Empirical Analysis of the PVAR

#### 4.2.1. Stationarity Analysis

The stationarity analysis of panel data is the basis for subsequent model estimation and impulse response, which can solve the spurious regression problem caused by unit root. In this study, after eliminating the effect of heteroskedasticity by taking the logarithm of the original data, the results of the ecological environment (ln*EE*) and urban resilience (ln*UR*) panel data were tested for unit root. We choose the LLC test, IPS test, and ADF–Fisher test to perform the smoothness analysis using Stata 16 software (StataCorp, College Station, TX, USA). As shown in Table 4, the variables ln*EE* and ln*UR* have a *p*-value of 0.000 on all three tests, indicating that both variables significantly reject the original hypothesis at the 1% level. Meanwhile, the ln*EE* and ln*UR* variables are both stationary series and passed the stationarity analysis.

#### 4.2.2. Lag Order Test

Under the premise that the panel data are stable, it is also necessary to determine the optimal lag order of the model. We comprehensively consider the results of the AIC criterion, the BIC criterion, and the HQIC criterion (Table 5). The results show that the optimal order of AIC and HQIC statistics is 5, and the optimal order of BIC statistic is 3. When the test results are inconsistent, the results based on the BIC/HQIC criteria are generally better than the AIC criterion [52,53], so the optimal lag order of the PVAR in this paper is chosen as 5.

#### 4.2.3. GMM of the PVAR

Before model estimation, we adopted the Granger causality test to verify that the ln*EE* and ln*UR* variables have a bidirectional causal relationship with a synergistic association between them. Based on this, we applied the panel generalized moment estimation (GMM) method to explore the interaction and the response mechanism between the EE and UR. The estimation results are shown in Table 6.

(1) L1_ln*EE* and lag 5 have significant positive effects on the current period. The coefficients of 0.484 and 0.227 indicate that the EE has a significant self-enhancement mechanism, and the enhancement effect gradually decreases with positive cumulative effects and inertia trends. The process of ecological restoration and treatment is often difficult, costly, time-consuming, and difficult to achieve immediate results with, so it needs to follow the laws of nature and economics: gradual progress and continuous investment. At the same time, coordinating and promoting the construction and development of the EE is not the task of a single region. All cities, organizations, and individuals in the basin are an important part. The Yangtze River Economic Belt should build a diversified ecological compensation mechanism around the life community of mountains, rivers, forests, fields, lakes, and grasses to build a solid ecological protection embankment for the Yangtze River Economic Belt, so as to ensure that the progressive effect of the EE can be given full play.

(2) L1_ln*UR* and L5_ln*UR* had a positive and significant effect on the current period, while L3_ln*UR* had a negative and significant effect on the current period, and their effect coefficients were 0.728, 0.175, and −0.257, respectively. This shows that the effect of the UR on itself has the characteristics of promotion–inhibition–promotion. The early and recent development of the UR has a divergent promotion effect on itself, while the mid-term development has a convergent inhibitory effect on itself. The reason may be that in the early stage, under the action of multi-faceted measures such as industrial structure adjustment, defense engineering construction, and the establishment of urban resilience culture, it played a short-term incentive and improvement role on the urban resilience level. However, in the middle stage, under the continuous effect of different strength drivers, conflicts and imbalances arise within the system and produce large fluctuations, which inhibit the performance of urban resilience. With the mutual adaptation of the various subsystems of urban resilience, they gradually show the coordination of system operation on the whole, effectively increase the function of the city, and promote the positive improvement of the level of the UR.

(3) L2_ln*EE* and L3_ln*EE* had a significant impact on the level of urban resilience, firstly negative and then positive, with the effect coefficients of −0.149 and 0.108, respectively, indicating that the UR has a dynamic characteristic of first inhibiting and then promoting the development of the EE. In the long run, the UR is the key to improving the ecological environment. The reason is that the activities of the UR play a restraining effect on the development of the EE in the early stage to varying degrees. The UR has gradually produced positive incentives for the EE, such as efficient allocation of ecological resources, scientific and technological innovation to promote green transformation, reasonable planning of ecological space, and continuous improvement of environmental awareness. This positive effect can alleviate the burden of environmental carrying capacity, optimize the rational layout of cities and ecological spaces, build collaborative governance of humanistic systems and ecosystems, and achieve new kinetic energy for green development. 

(4) L2_ln*UR* and L3_ln*UR* had a significant negative impact on the ecological environment at first, and then a positive one, and their effect coefficients were −0.456 and 0.418, respectively, indicating that the impact of the EE on the level of the UR showed the characteristics of first inhibition and then promotion. The EE has a convergent inhibitory effect on the level of the UR in the early development process, while it has a divergent promoting effect on the level of UR in the recent development process. In the long run, the EE is an important support for the construction of resilient cities. The reason for the emergence of first inhibition and then promotion is that the UR belongs to systematic and comprehensive construction, which is influenced by the concept of green development of the EE in the early stage. Influenced by the concept of the green development of the ecological environment in the early stage, economic development and infrastructure construction will inevitably be restricted to a certain extent. With the construction of the EE value-added function, the efficacy of the environmental condition guarantee and ecological response mechanism, which bring material bases and value elements for the UR, forms healthy urban development modes and enhances the UR.

#### 4.2.4. Impulse Response Analysis

**Figure 5:**
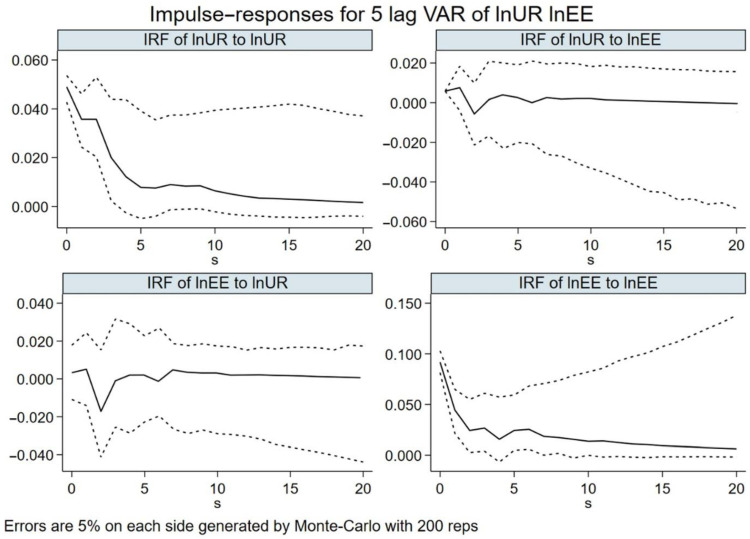
Impulse response of ecological environment and urban resilience. Note: The solid line represents the impulse response curve; and the dotted line represents the 95% confidence interval range.

To further explore the dynamic effects of a shock of a standard deviation on the endogenous variables in the current and future periods, we used impulse response functions to reflect the degree of variable response to random disturbance shocks. Through 200 random Monte Carlo simulations, the dynamic interactive response trend of the EE and UR in the next 20 periods was analyzed. The results were obtained as shown in Figure 5, with the horizontal axis representing the number of response periods and the vertical axis representing the magnitude of response.

The ln*EE* of provinces and cities in the Yangtze River Economic Belt shows a significant positive impulse response to its own one standard deviation shock, reaching a maximum in the same period, with obvious fluctuations in the first five periods, and a gentle downward trend and gradual convergence after the sixth period. The ln*UR* also shows a significant positive response in the face of its own one standard deviation shock, with the maximum value in the first period, a large fluctuation in the first three periods, and a decreasing positive impulse response after the fourth period. The above results are consistent with the GMM results, indicating that both the EE and UR have a self-enhancement effect and path-dependent characteristics, and the response has the characteristic of decreasing over time. This is consistent with the reality of ecological environment development and urban resilience construction. In addition to giving full play to the self-enhancement mechanism of both, we should actively adjust the development strategy and actively improve the efficiency of resource allocation to slow down the decreasing rate of response.

The ln*EE* variable faces a standard deviation ln*UR* shocks; the EE shows “N” shaped fluctuations in the first three periods, with a slowly rising positive response in the same period and lag 1, reaching its highest point in lag 1, followed by a rapid decline in lag 2, showing a significant negative effect, a rapid rebound in lag 3 and lag 4, and a gradual positive convergence in the later period. The ln*UR* variable faces one standard deviation of ln*EE* shock’ the UR has a smaller positive effect in the same period, and lag 1 continues to rise, and then rapidly declines to lag 2. Although lag 3 reaches a larger rebound, it still cannot offset the negative effect brought by lag 2 at this time; lag 4 and lag 5 continue to rise, and then gradually stabilize and converge positively. Overall, the impacts of the UR on the EE and the EE on the UR show a positive impulse effect on each other, which demonstrate that the overall EE and UR in the Yangtze River Economic Belt are mutually reinforcing, and there is a mechanism of mutual support, mutual influence, and interactive response between the two. However, this mechanism was not stable in the early stage, showing a short-term interactive stress effect. This may be due to the high vulnerability of the ecological environment development in the Yangtze River Economic Belt and the overall low level of the UR, which produced an obvious inhibitory effect among the systems in the early stage. At the same time, the construction and initiatives related to the EE and UR of each province and city in the Yangtze River Economic Belt vary in strength, with obvious regional differences, thus limiting the overall effect of the EE and UR, resulting in the negative driving effect between them in the early stage.

#### 4.2.5. Variance Decomposition Model

Forecast Error Variance Decomposition (FEVD) is a method to evaluate the degree of influence of mutual shocks between endogenous variables. That is, it can measure the mutual contribution between ln*EE* and ln*UR* in the forecast period and examine the degree of importance between variables [54]. The variance decomposition can quantitatively reflect the difference in the intensity of the mutual impact between the EE and UR, and the results are shown in Table 7. The degrees of influence of the EE and UR on their own shocks in period 1 are 99.9% and 100%, respectively, and in period 20, the degree of contribution of the EE to its own is still as high as 96.1%, while the UR has rapidly decreased to 93.0%. The results continue to confirm that the EE and UR rely on their own inertia development and have path-dependent characteristics. The contribution of the EE to the UR and the UR to the EE are 0.1% and 0.3% in period 1, respectively, and continue to increase afterwards, reaching 3.9% and 7.0% in period 20, respectively. This indicates that there is a degree of contribution between both the EE and UR, and the degree of contribution and explanatory power of the UR to EE is greater. In the interaction between the EE and the UR, the UR plays a more obvious role in promotion, which is of great significance to the improvement of ecosystems and overall functions. In the process of focusing on resilient city construction in the Yangtze River Economic Belt, we simultaneously adhere to the concept of ecological priority and green transformation, strengthen regional synergy, enhance the concept of interconnection and mutual protection, and form a governance pattern of the UR based on protecting and restoring the Yangtze River ecological environment, so as to achieve sustainable optimization of the EE in the Yangtze River Economic Belt.

## 5. Discussion

This study takes 11 provinces and cities in the Yangtze River Economic Belt as the research object. Firstly, the ecological environment index and urban resilience index are measured and evaluated by using the entropy method. Secondly, the coupling coordination degree model is constructed to explore the coupling mechanism of ecological environment and urban resilience. Finally, the PVAR is used to investigate and reveal the interactive response relationship between ecological environment and urban resilience.

In the existing literature, there are few studies on the coordination of ecological environment and urban resilience, and there is still no relevant research to discuss the interaction process between ecological environment and urban resilience. Considering that the Yangtze River Economic Belt has always emphasized ecological priority and green development, as well as the construction of resilient cities, we take the Yangtze River Economic Belt as the research object that is more in line with our research theme. Under the theme of coordinated research on ecological environment and urban resilience, we have deeply explored and solved the following four questions: What is the development level of the ecological environment system and urban resilience system in the Yangtze River Economic Belt? How good is the coupling coordination degree between ecological environment and urban resilience? How do the ecological environment and urban resilience interact and how do the specific interactions change? Based on the sustainable development theory, we innovatively construct a coordination mechanism framework for ecological environment and urban resilience (Figure 1). According to its development degree, coordination degree, and sustainability degree, systematic evaluation, coupling coordination calculation, and PVAR estimation are carried out, respectively, so as to realize the discussion of the coordination relationship between ecosystem and urban system. We embody the bottom-line thinking of protecting the ecological environment and the rational development thinking of building a resilient city in the Yangtze River Economic Belt, thus emphasizing the positive and promoting relationship of interdependence between humans and nature, so as to achieve harmonious development between systems.

The temporal and spatial characteristics of cities in the Yangtze River Economic Belt are significantly different, and the differences between regions are large. The characteristic of “strong in the east and weak in the west” is obvious, and the overall effect of the basin is weak. Therefore, in order to realize a virtuous circle in which the ecological environment and urban resilience of the Yangtze River Economic Belt coordinate and promote each other, a differentiated improvement strategy should be implemented to strengthen the coordinated development of the upper, middle, and lower reaches of the Yangtze River. We must grasp the important opportunities of ecological environmental protection to promote the construction of resilient cities. In this regard, we will take into account the geospatial structure and regional resource endowments and seek a synergistic path to improve the ecological environment and urban resilience. Based on a global understanding of the development of the Yangtze River Economic Belt, we will strengthen the policy synergy between the provinces and cities in the region. We suggest creating a model for the synergistic management of ecological environment and urban resilience in mega cities, with Shanghai, Jiangsu, Hubei, and Chongqing as demonstration samples, giving full play to their radiation-driven role.

Our results show that although the coordination level between the ecological environment and the urban resilience system is high, the overall system tends to decline. The reason is that the system relationship of “one strong and one weak” leads to the lag of ecological environment or the lag of urban resilience. Therefore, the construction of development-oriented regions should be synchronized. On the basis of giving full play to the system advantages of different regions, we should focus on strengthening the design and development of the lagging system. The lower reaches of the Yangtze River should increase investment in ecological environment, aim at “carbon peaking and carbon neutrality”, cultivate low-carbon transformation of industrial structure, and upgrade science and technology innovation. We suggest improving the carrying capacity of resources and environment and achieving high-level development of the ecological environment. In the middle and upper reaches of Yangtze River, we will strengthen the emergency management system and enhance the ability of cities to carry out emergency operations efficiently. At the same time, this attaches great importance to the coordinated management of economic, social, ecological, infrastructure, and cultural systems and establishes an urban system integrating prevention, monitoring, emergency response, and restoration, so as to effectively resist the threat of various shocks.

We demonstrate the positive relationship between the ecological environment and urban resilience of the Yangtze River Economic Belt. Therefore, an interactive response mechanism between the ecological environment and urban resilience should be constructed to strengthen the ability to cope with early inhibitory effects and to play the supporting role of the ecological environment to urban resilience and the role of urban resilience to ecological environment enhancement (Figure 1). Among them, we focus on the driving effect of urban resilience on ecological environment, with Shanghai, Jiangsu, and Zhejiang as representatives of resilient cities, and play its demonstration and leading role for the middle and upper reaches of Yangtze River. Meanwhile, we establish the concept of green development, insist on green industry, green economy, green building, and green technology innovation. We believe it is important to weaken the interactive stress effect between early systems.

Our research only proves the coupling coordination relationship and interactive response mechanism of the ecological environment and urban resilience. We lack research on the influencing factors of the interaction between the two. Considering that the interaction has sensitive characteristics under different scale-dependent conditions, there will be regional differences in the degree and direction of the interaction response due to the heterogeneity of the scale. In addition, our research scale only considers the provincial level, and it is necessary to conduct extended research at different scales in the future. In particular, PVAR is currently the best way to check the relationship between two systems. However, if the number of indicators is too large, the number of PVAR structural equations will increase dramatically, which can easily exceed the degrees of freedom and data requirements. Therefore, when using the PVAR method, attention should be paid to the number of research indicators. Avoiding blindly pursuing the comprehensiveness of the indicator system magnifies the limitations of the PVAR method.

## 6. Conclusions

The ecological environment index and urban resilience index of the Yangtze River Economic Belt during the 2006–2020 period have obvious spatial and temporal divergent characteristics. In terms of time characteristics, various provinces and cities showed a fluctuating upward trend, and the overall development trend was good. Compared with the ecological environment index, the change in the urban resilience index is smaller, but the overall level is not high. The mean values of the two indices show significant regional differences, and the spatial characteristics of the ecological environment are “the lower reaches of the Yangtze River > the upper reaches of the Yangtze River > the middle reaches of the Yangtze River”. The overall spatial distribution pattern of urban resilience is “the lower reaches of the Yangtze River > the middle reaches of the Yangtze River > the upper reaches of the Yangtze River”.

The level of coordination between the ecological environment and urban resilience coupling coordination in the Yangtze River Economic Belt has steadily increased during the study period, and the coordination status is good, forming a spatial distribution pattern of “strong in the east—weak in the west”. The cities in the region are mainly at the basic coordination state or above, and the linkage between the two is becoming more and more obvious. The cities in the lower reaches of the Yangtze River are mainly characterized by ecological environment lag type. The cities in the middle and upper reaches of the Yangtze River are mainly characterized by urban resilience lag type.

The ecological environment and urban resilience in the Yangtze River Economic Belt have significant self-reinforcing mechanisms, and the strengthening effect gradually decreases with positive cumulative effects and inertia trends, in which the degree of attenuation of urban resilience to its own shock response is more obvious. Both the ecological environment and urban resilience have a first negative and then positive effect on the ecological environment, and the mechanism of their effects is positive in the long run. Additionally, in the 20th period, both of them are stable and positively converge. In the interaction between the ecological environment and urban resilience, urban resilience shows a more obvious promoting effect on the ecological environment.

## Figures and Tables

**Table 1:** Ecological Environment and Urban Resilience Evaluation Index System.

Target	Dimensions	Indicators	Properties	Weights
Ecological Environment	Pressure (0.27)	Natural disaster damage	−	0.08
		COD emissions in industrial wastewater per unit of GDP	−	0.07
		Industrial sulfur dioxide emissions per unit of GDP	−	0.05
		Fertilizer application amount per unit of agricultural output value	−	0.07
	State (0.32)	Excellent air quality rate	+	0.08
		Water resources per capita	+	0.09
		Forest cover rate	+	0.09
		Green space per capita	+	0.07
	Response (0.41)	Soil erosion control area	+	0.1
		Energy conservation and environmental protection expenditure as a percentage of GDP	+	0.08
		Municipal domestic waste removal volume	+	0.14
		Municipal sewage treatment rate	+	0.08
Urban Resilience	Economic resilience (0.24)	GDP per capita	+	0.04
		Production of tertiary sector in GDP	+	0.04
		Per capita disposable income of urban residents	+	0.06
		Local public finance revenue	+	0.04
		Actual amount of foreign capital used in the year	+	0.04
		Growth of in Fixed assets investment per capita	+	0.02
	Social resilience (0.19)	Population density	+	0.04
		Urban registered unemployment rate	-	0.03
		Number of beds in health care institutions	+	0.03
		Regional urbanization rate	+	0.03
		Share of social security expenditure in fiscal expenditure	+	0.03
		Pension insurance coverage rate	+	0.03
	Ecological resilience (0.12)	Greening coverage rate of built-up areas	+	0.02
		Forest cover rate	+	0.02
		Industrial smoke (powder) dust emissions per unit of GDP	−	0.02
		Industrial wastewater emissions per unit of GDP	−	0.02
		Harmless treatment rate of domestic waste	+	0.02
		Comprehensive utilization rate of industrial solid waste	+	0.03
	Infrastructure resilience (0.19)	Number of public buses per 10,000 people	+	0.02
		Number of Internet broadband access users	+	0.04
		Electricity consumption per capita	+	0.05
		Daily domestic water consumption per catipa	+	0.02
		Total city gas supply	+	0.04
		Urban road area per capita	+	0.02
	Cultural resilience (0.26)	Proportion of public cultural expenditure to local budget expenditure	+	0.03
		Number of books in public libraries per 100 people	+	0.09
		Number of people employed in culture, sports and entertainment per 10,000 people	+	0.03
		Regional cable radio and television subscribers	+	0.04
		Number of college students in general higher education institutions	+	0.03
		The proportion of the population with a college degree and above in the population over the age of 15	+	0.05

**Table 2:** Coordination level classification of ecological environment and urban resilience.

*D*	Type	Relative Size of UR and EE	Feature	Code
0 ≤ *D* < 0.3	Severe disorder	UR−EE > 0.1	Severe disorder—The EE lags behind, and the system tends to decline	I1
		0 ≤ |UR−EE| ≤ 0.1	Severe disorder—The system tends to be optimized	I2
		EE−UR > 0.1	Severe disorder—The UR lags behind, and the system tends to decline	I3
0.3 ≤ *D* < 0.5	Basic disorder	UR−EE > 0.1	Basic disorder—The EE lags behind, and the system tends to decline	II1
		0 ≤ |UR−EE| ≤ 0.1	Basic disorder—The system tends to be optimized	II2
		EE−UR > 0.1	Basic disorder—The UR lags behind, and the system tends to decline	II3
0.5 ≤ *D* < 0.8	Basic coordination	UR−EE > 0.1	Basic coordination—The EE lags behind, and the system tends to decline	III1
		0 ≤ |UR−EE| ≤ 0.1	Basic coordination—The system tends to be optimized	III2
		EE−UR > 0.1	Basic coordination—The UR lags behind, and the system tends to decline	III3
0.8 ≤ *D* < 1	Senior coordination	UR−EE > 0.1	Senior coordination—The EE lags behind, and the system tends to decline	IV1
		0 ≤ |UR−EE| ≤ 0.1	Senior coordination—The system tends to be optimized	IV2
		EE−UR > 0.1	Senior coordination—The UR lags behind, and the system tends to decline	IV3

**Table 3:** Coupling coordination degree between the EE and UR.

Year	Shanghai	Jiangsu	Zhejiang	Anhui	Jiangxi	Hubei	Hunan	Chongqing	Sichuan	Guizhou	Yunnan
2006	0.80	0.76	0.77	0.55	0.56	0.62	0.57	0.55	0.62	0.43	0.53
2007	0.81	0.77	0.77	0.54	0.56	0.61	0.55	0.52	0.59	0.44	0.52
2008	0.81	0.80	0.78	0.55	0.57	0.62	0.59	0.56	0.63	0.42	0.54
2009	0.83	0.80	0.80	0.54	0.58	0.62	0.58	0.57	0.62	0.48	0.54
2010	0.78	0.80	0.80	0.58	0.58	0.62	0.59	0.61	0.60	0.47	0.53
2011	0.78	0.80	0.80	0.58	0.59	0.61	0.58	0.66	0.65	0.44	0.52
2012	0.78	0.78	0.78	0.59	0.59	0.61	0.58	0.65	0.64	0.49	0.53
2013	0.75	0.81	0.79	0.61	0.58	0.66	0.59	0.65	0.66	0.50	0.54
2014	0.75	0.82	0.81	0.61	0.58	0.65	0.62	0.67	0.63	0.50	0.52
2015	0.73	0.79	0.79	0.59	0.60	0.61	0.63	0.65	0.62	0.51	0.50
2016	0.74	0.79	0.81	0.60	0.56	0.62	0.66	0.67	0.61	0.52	0.52
2017	0.77	0.79	0.81	0.61	0.58	0.62	0.65	0.65	0.62	0.53	0.53
2018	0.74	0.75	0.78	0.60	0.59	0.65	0.62	0.67	0.66	0.54	0.56
2019	0.71	0.74	0.79	0.59	0.57	0.66	0.66	0.64	0.69	0.54	0.51
2020	0.70	0.74	0.78	0.59	0.62	0.62	0.69	0.70	0.65	0.53	0.54
Average value	0.77	0.78	0.79	0.58	0.58	0.63	0.61	0.63	0.63	0.49	0.53

**Table 4:** Variable Stationarity Analysis.

Variables	LLC Statistic	IPS Statistics	ADF-Fisher Statistics
ln*UR*	−5.0286 *** (0.000)	−4.1691 *** (0.000)	6.4645 *** (0.000)
ln*EE*	−4.8163 *** (0.000)	−3.4650 *** (0.000)	3.0253 *** (0.001)

Note: *** indicates that the variable significantly rejects the null hypothesis at the 1% level.

**Table 5:** Selection of the optimal lag order.

lag	AIC	BIC	HQIC
1	−2.41559	−1.87689	−2.19669
2	−3.76758	−3.11240	−3.50135
3	−4.10648	−3.32088 *	−3.78742
4	−4.19745	−3.26455	−3.81906
5	−4.28430 *	−3.18334	−3.83885 *

Note: * represents the optimal lag order of the model.

**Table 6:** Estimation results of PVAR based on the GMM method.

Variables	Explained Variable ln*UR*	Variables	Explained Variable ln*EE*
L1_ln*UR*	0.728 *** (6.05)	L1_ln*UR*	0.072 (0.30)
L2_ln*UR*	0.206 (1.57)	L2_ln*UR*	−0.456 ** (−1.07)
L3_ln*UR*	−0.257 *** (−2.84)	L3_ln*UR*	0.418 * (2.24)
L4_ln*UR*	−0.073 (−0.59)	L4_ln*UR*	0.074 (0.46)
L5_ln*UR*	0.175 ** (2.08)	L5_ln*UR*	0.069 (0.45)
L1_ln*EE*	0.019(0.28)	L1_ln*EE*	0.484 *** (3.66)
L2_ln*EE*	−0.149 ** (−2.51)	L2_ln*EE*	0.028 (0.28)
L3_ln*EE*	0.108 * (1.78)	L3_ln*EE*	0.166 (1.50)
L4_ln*EE*	0.028 (0.42)	L4_ln*EE*	−0.121 (−1.10)
L5_ln*EE*	0.003 (0.05)	L5_ln*EE*	0.227 ** (2.07)

Note: ***, **, and * indicate significance levels of 1%, 5%, and 10%, respectively. L1, L2, L3, L4, and L5 represent lags 1, 2, 3, 4, and 5, respectively. Values in parentheses are z-statistics.

**Table 7:** Prediction error variance decomposition results (%).

	ln*EE*		ln*UR*	
*s*	ln*EE*	ln*UR*	ln*UR*	ln*EE*
1	99.9	0.3	100.0	0.1
5	97.2	3.5	96.5	2.8
10	96.8	5.1	94.9	3.2
15	96.4	6.3	93.7	3.6
20	96.1	7.0	93.0	3.9

Note: The table presents the variance decomposition results for periods 1, 5, 10, 15, and 20 only. *s* denotes the number of forecast periods.

## Data Availability

Not applicable.
